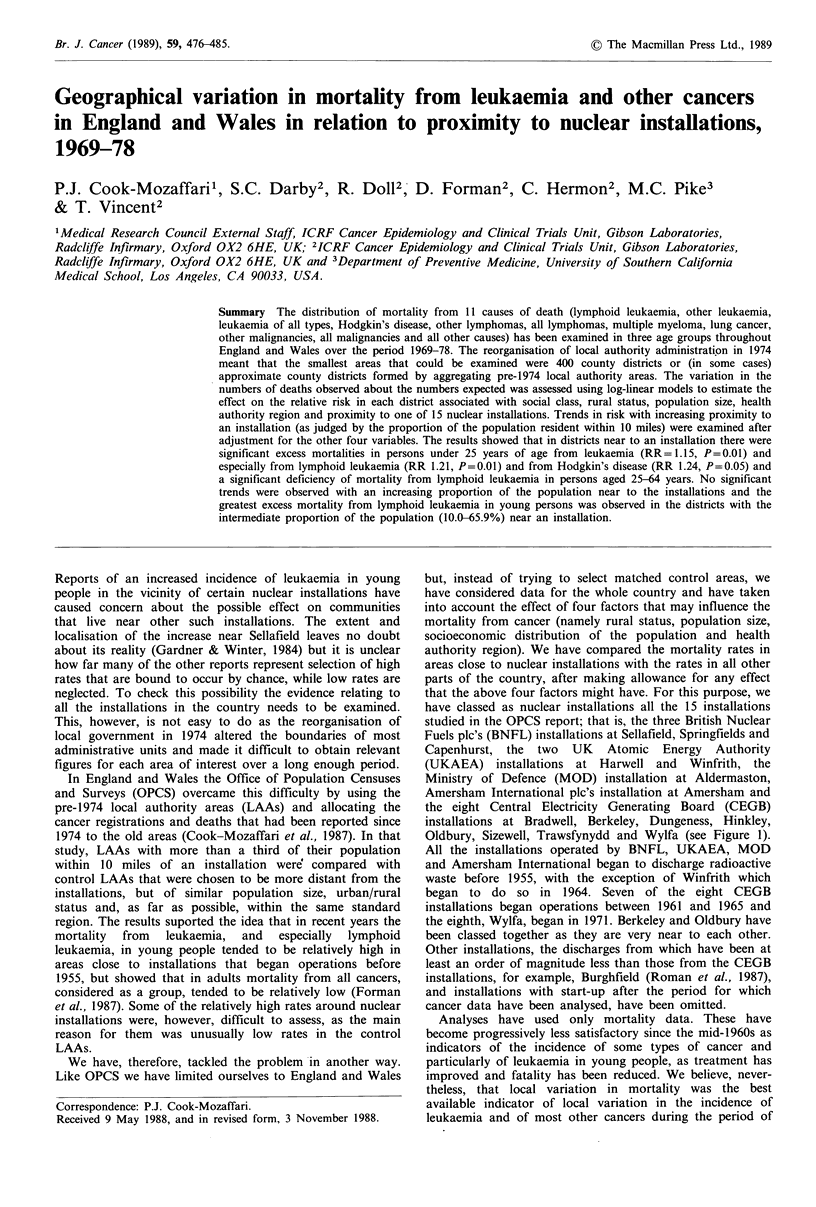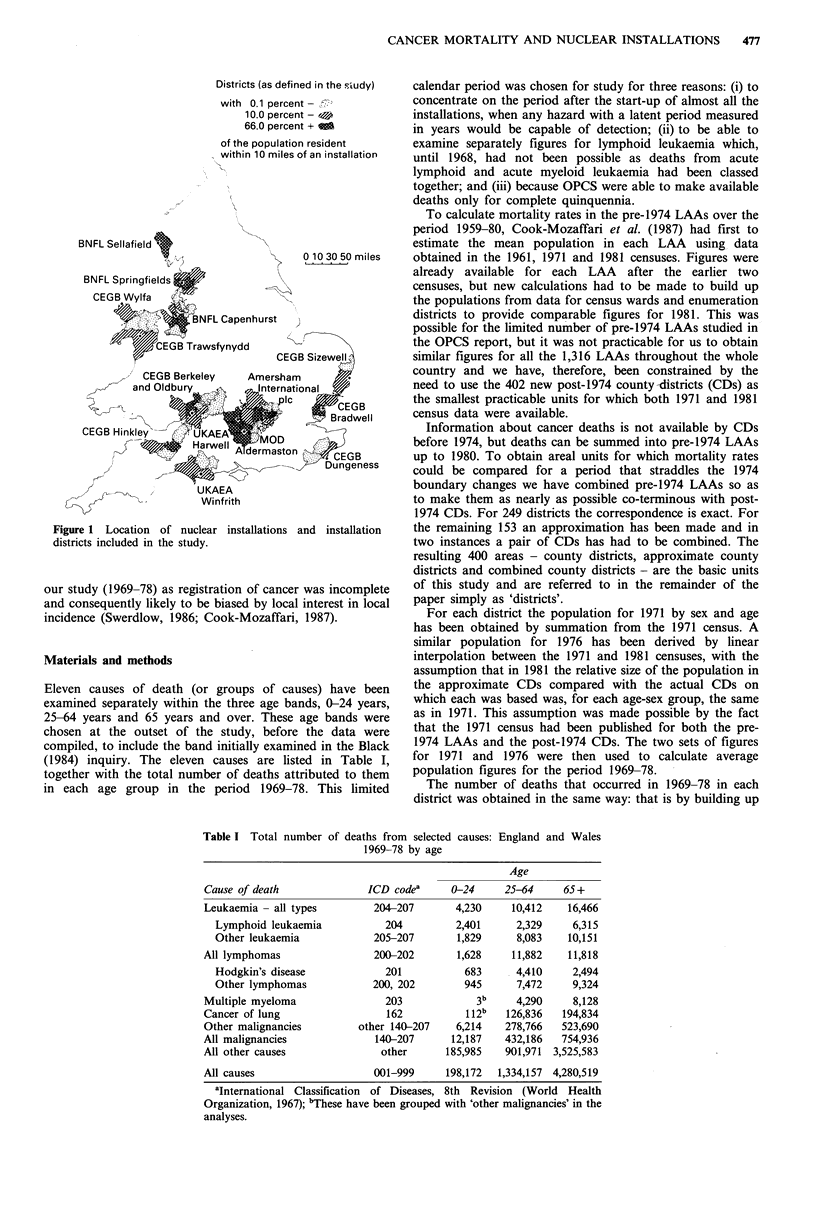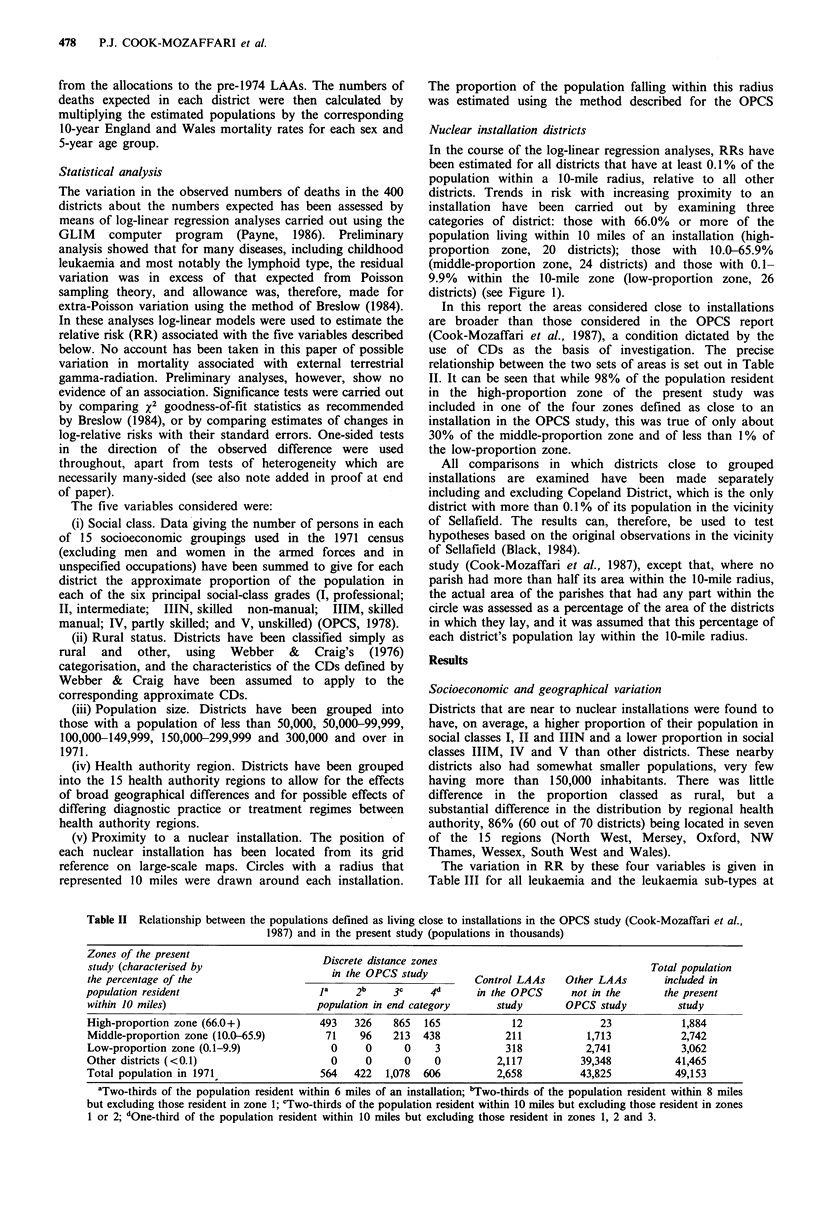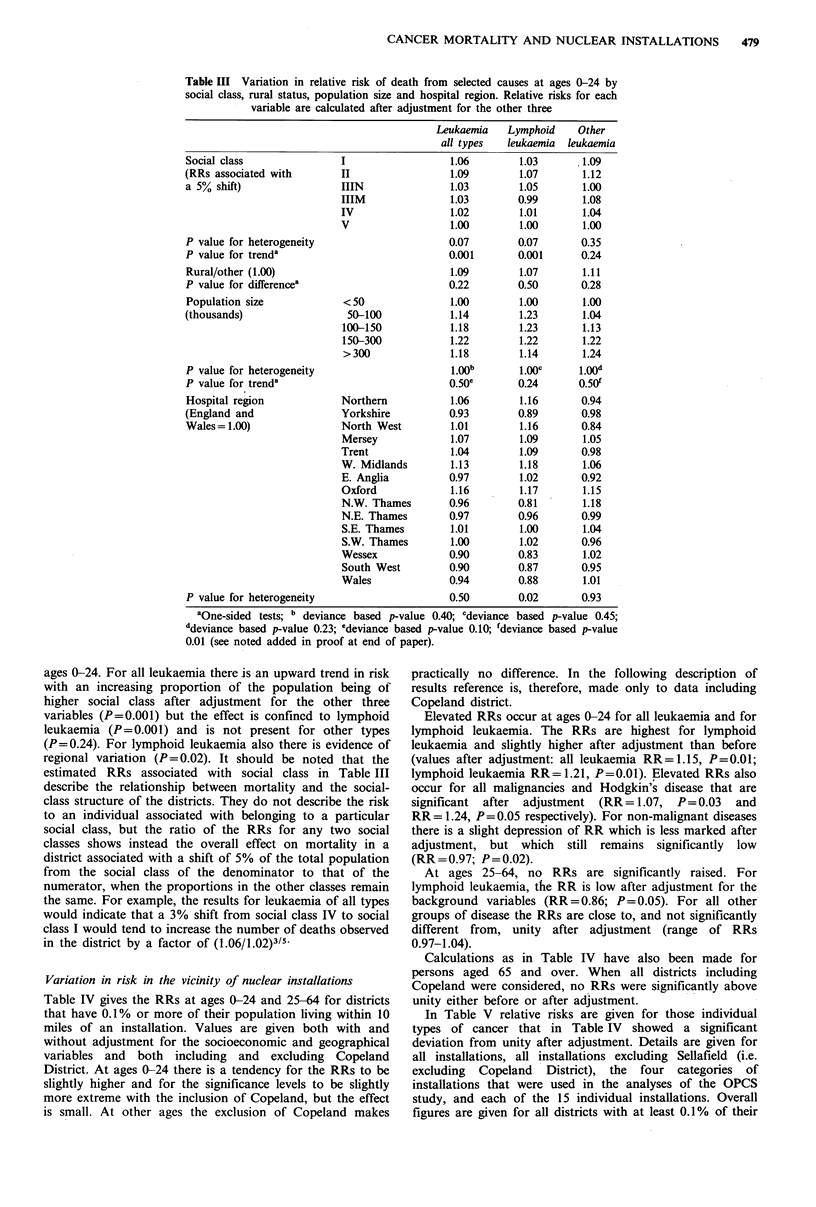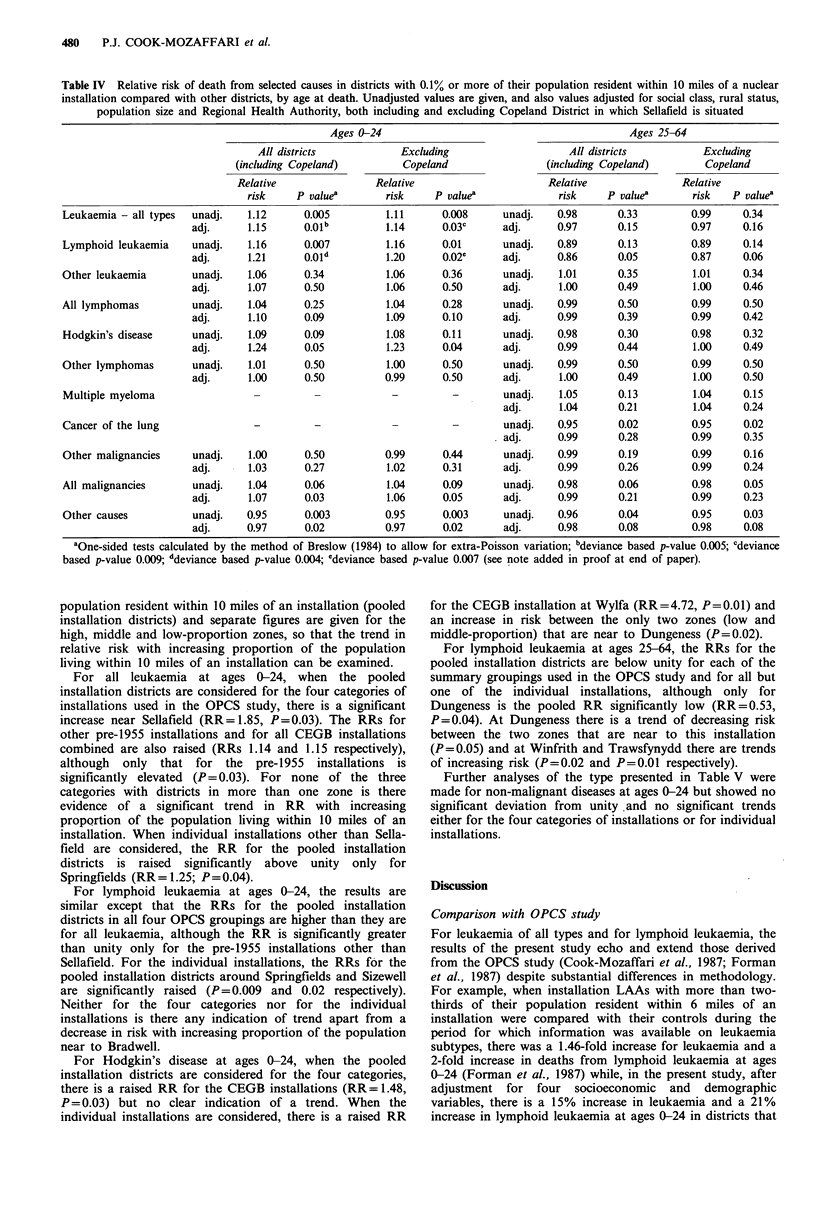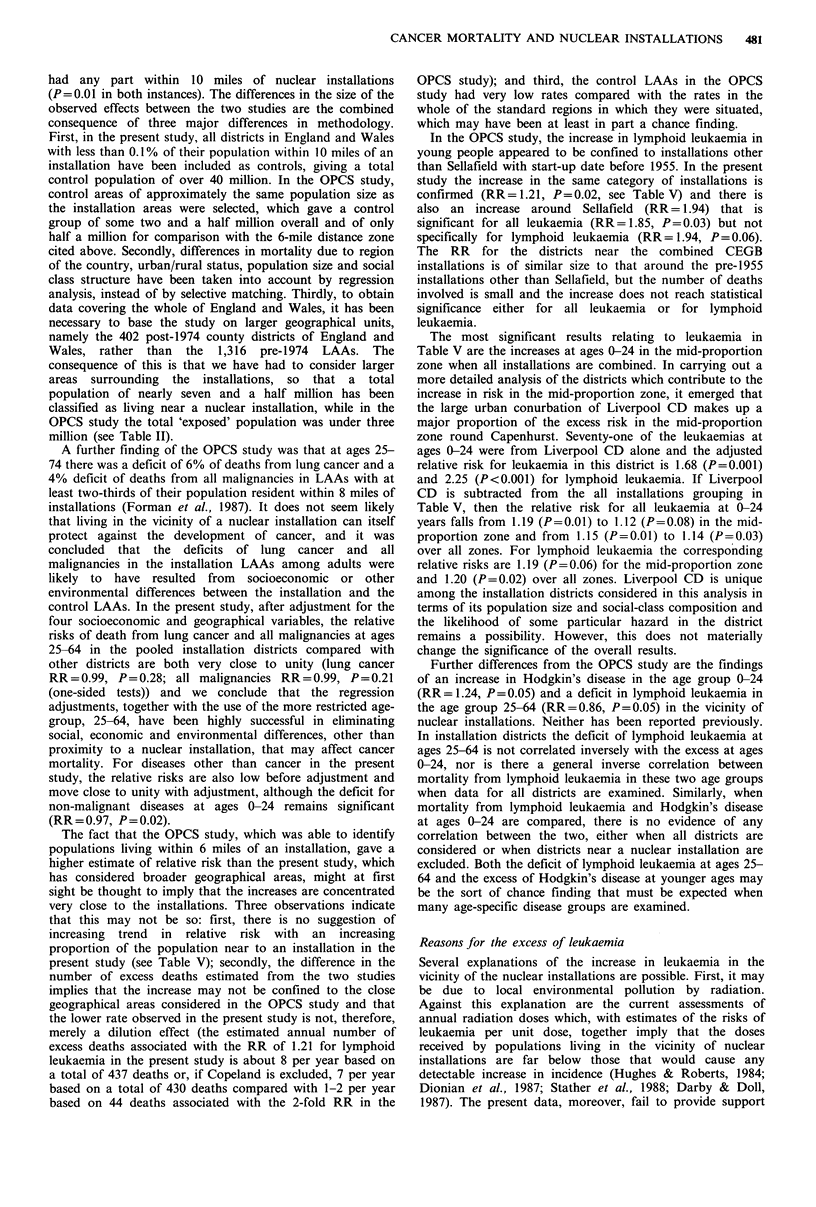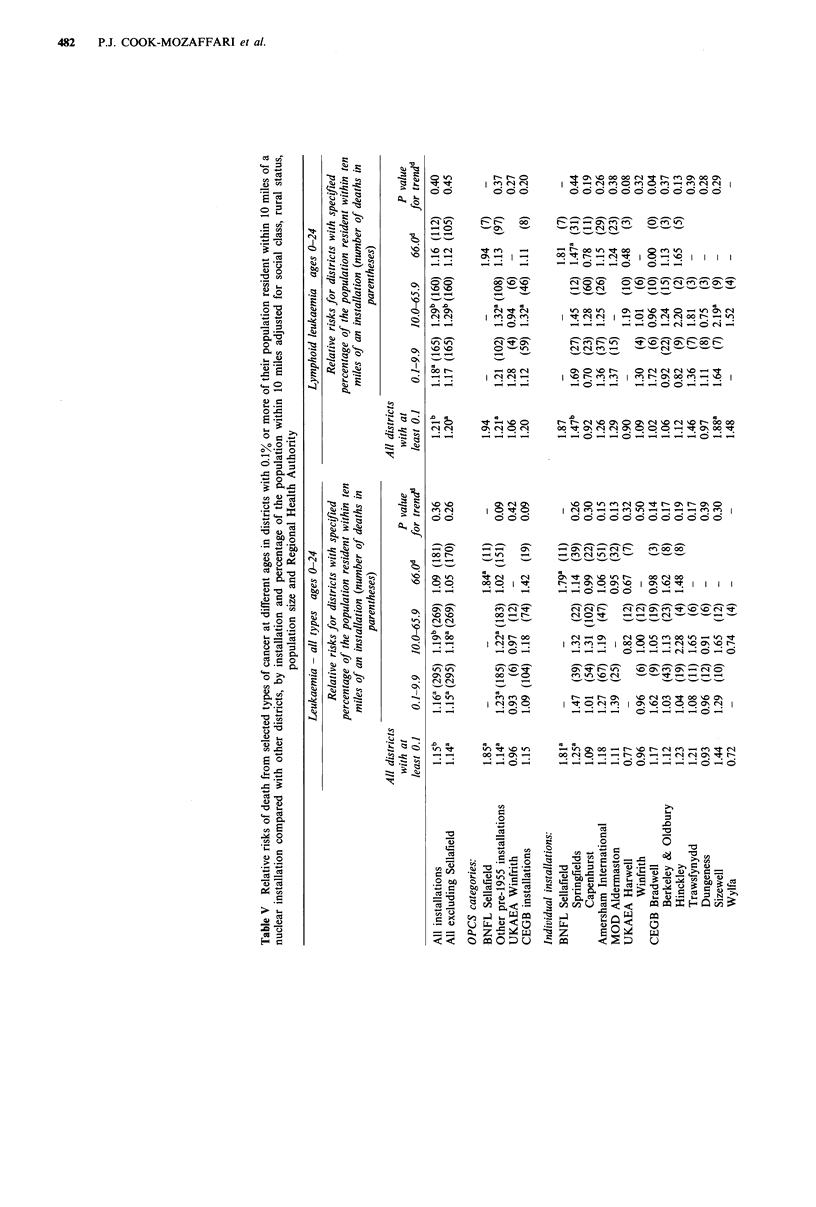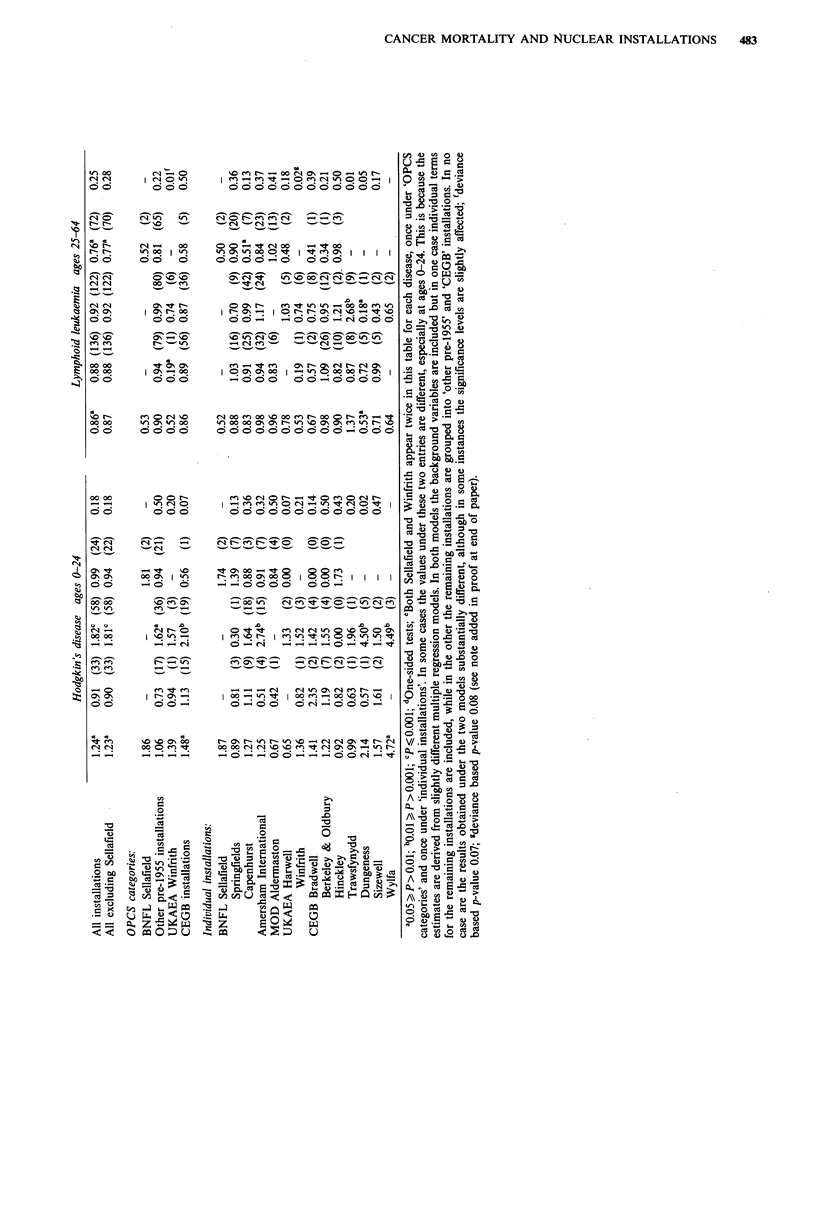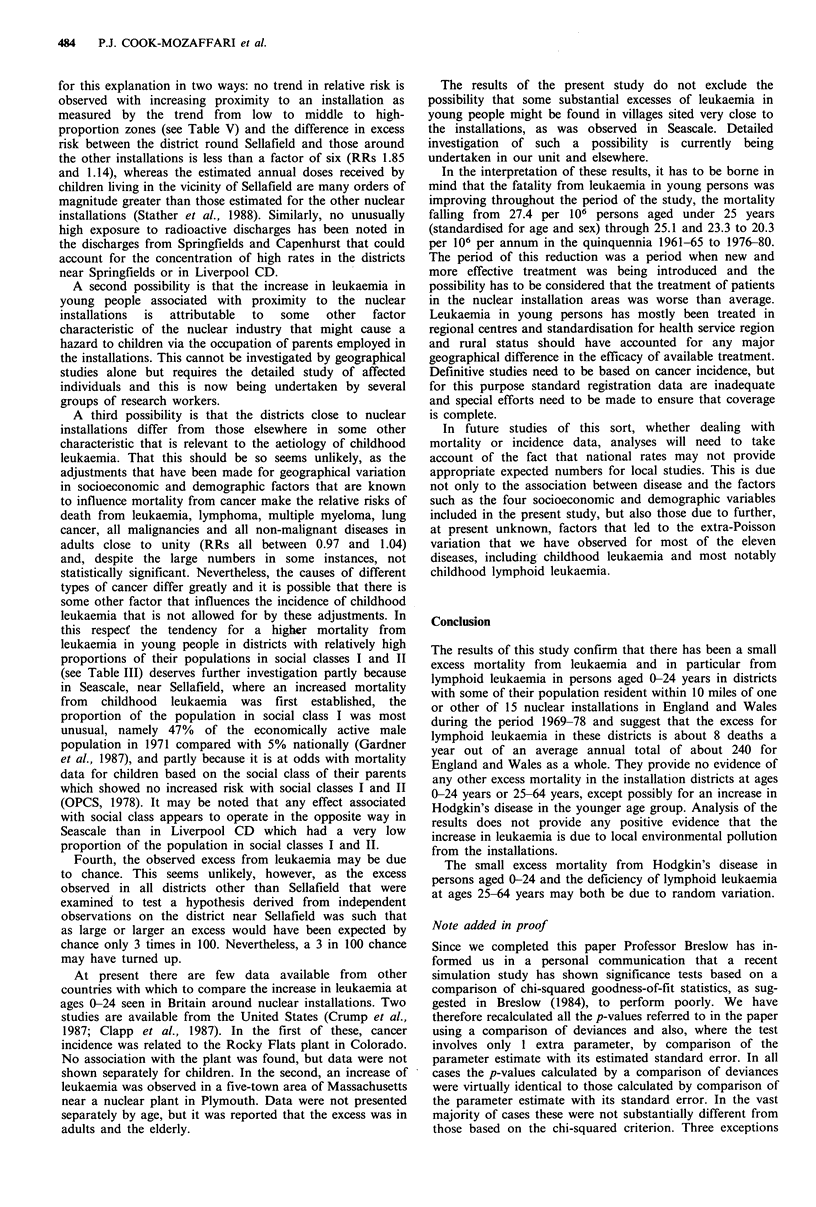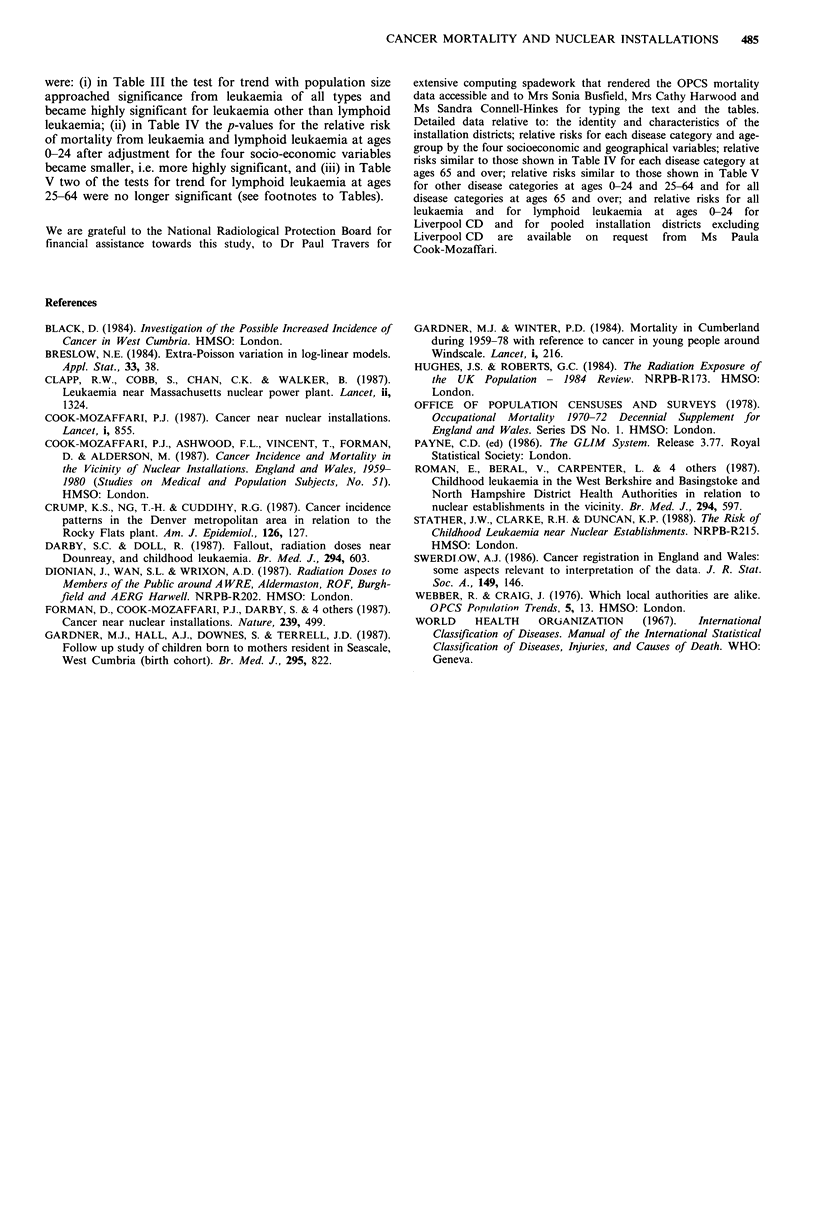# Geographical variation in mortality from leukaemia and other cancers in England and Wales in relation to proximity to nuclear installations, 1969-78.

**DOI:** 10.1038/bjc.1989.99

**Published:** 1989-03

**Authors:** P. J. Cook-Mozaffari, S. C. Darby, R. Doll, D. Forman, C. Hermon, M. C. Pike, T. Vincent

**Affiliations:** ICRF Cancer Epidemiology and Clinical Trials Unit, Gibson Laboratories, Radcliffe Infirmary, Oxford, UK.

## Abstract

The distribution of mortality from 11 causes of death (lymphoid leukaemia, other leukaemia, leukaemia of all types, Hodgkin's disease, other lymphomas, all lymphomas, multiple myeloma, lung cancer, other malignancies, all malignancies and all other causes) has been examined in three age groups throughout England and Wales over the period 1969-78. The reorganisation of local authority administration in 1974 meant that the smallest areas that could be examined were 400 county districts or (in some cases) approximate county districts formed by aggregating pre-1974 local authority areas. The variation in the numbers of deaths observed about the numbers expected was assessed using log-linear models to estimate the effect on the relative risk in each district associated with social class, rural status, population size, health authority region and proximity to one of 15 nuclear installations. Trends in risk with increasing proximity to an installation (as judged by the proportion of the population resident within 10 miles) were examined after adjustment for the other four variables. The results showed that in districts near to an installation there were significant excess mortalities in persons under 25 years of age from leukaemia (RR = 1.15, P = 0.01) and especially from lymphoid leukaemia (RR 1.21, P = 0.01) and from Hodgkin's disease (RR 1.24, P = 0.05) and a significant deficiency of mortality from lymphoid leukaemia in persons aged 25-64 years. No significant trends were observed with an increasing proportion of the population near to the installations and the greatest excess mortality from lymphoid leukaemia in young persons was observed in the districts with the intermediate proportion of the population (10.0-65.9%) near an installation.


*Br. J. Cancer* (1989), **59**, 476–485.
© The Macmillan Press Ltd., 1989

### Geographical variation in mortality from leukaemia and other cancers in England and Wales in relation to proximity to nuclear installations, 1969–78

P.J. Cook-Mozaffari[1], S.C. Darby[2], R. Doll[2], D. Forman[2], C. Hermon[2], M.C. Pike[3] & T. Vincent[2]

[1]*Medical Research Council External Staff, ICRF Cancer Epidemiology and Clinical Trials Unit, Gibson Laboratories, Radcliffe Infirmary, Oxford OX2 6HE, UK;* [2]*ICRF Cancer Epidemiology and Clinical Trials Unit, Gibson Laboratories, Radcliffe Infirmary, Oxford OX2 6HE, UK and* [3]*Department of Preventive Medicine, University of Southern California Medical School, Los Angeles, CA 90033, USA.*

**Summary** The distribution of mortality from 11 causes of death (lymphoid leukaemia, other leukaemia, leukaemia of all types, Hodgkin's disease, other lymphomas, all lymphomas, multiple myeloma, lung cancer, other malignancies, all malignancies and all other causes) has been examined in three age groups throughout England and Wales over the period 1969–78. The reorganisation of local authority administration in 1974 meant that the smallest areas that could be examined were 400 county districts or (in some cases) approximate county districts formed by aggregating pre-1974 local authority areas. The variation in the numbers of deaths observed about the numbers expected was assessed using log-linear models to estimate the effect on the relative risk in each district associated with social class, rural status, population size, health authority region and proximity to one of 15 nuclear installations. Trends in risk with increasing proximity to an installation (as judged by the proportion of the population resident within 10 miles) were examined after adjustment for the other four variables. The results showed that in districts near to an installation there were significant excess mortalities in persons under 25 years of age from leukaemia (RR = 1.15, $P = 0.01$) and especially from lymphoid leukaemia (RR 1.21, $P = 0.01$) and from Hodgkin's disease (RR 1.24, $P = 0.05$) and a significant deficiency of mortality from lymphoid leukaemia in persons aged 25–64 years. No significant trends were observed with an increasing proportion of the population near to the installations and the greatest excess mortality from lymphoid leukaemia in young persons was observed in the districts with the intermediate proportion of the population (10.0–65.9%) near an installation.

Reports of an increased incidence of leukaemia in young people in the vicinity of certain nuclear installations have caused concern about the possible effect on communities that live near other such installations. The extent and localisation of the increase near Sellafield leaves no doubt about its reality (Gardner & Winter, 1984) but it is unclear how far many of the other reports represent selection of high rates that are bound to occur by chance, while low rates are neglected. To check this possibility the evidence relating to all the installations in the country needs to be examined. This, however, is not easy to do as the reorganisation of local government in 1974 altered the boundaries of most administrative units and made it difficult to obtain relevant figures for each area of interest over a long enough period.

In England and Wales the Office of Population Censuses and Surveys (OPCS) overcame this difficulty by using the pre-1974 local authority areas (LAAs) and allocating the cancer registrations and deaths that had been reported since 1974 to the old areas (Cook-Mozaffari *et al.*, 1987). In that study, LAAs with more than a third of their population within 10 miles of an installation were compared with control LAAs that were chosen to be more distant from the installations, but of similar population size, urban/rural status and, as far as possible, within the same standard region. The results suported the idea that in recent years the mortality from leukaemia, and especially lymphoid leukaemia, in young people tended to be relatively high in areas close to installations that began operations before 1955, but showed that in adults mortality from all cancers, considered as a group, tended to be relatively low (Forman *et al.*, 1987). Some of the relatively high rates around nuclear installations were, however, difficult to assess, as the main reason for them was unusually low rates in the control LAAs.

We have, therefore, tackled the problem in another way. Like OPCS we have limited ourselves to England and Wales but, instead of trying to select matched control areas, we have considered data for the whole country and have taken into account the effect of four factors that may influence the mortality from cancer (namely rural status, population size, socioeconomic distribution of the population and health authority region). We have compared the mortality rates in areas close to nuclear installations with the rates in all other parts of the country, after making allowance for any effect that the above four factors might have. For this purpose, we have classed as nuclear installations all the 15 installations studied in the OPCS report; that is, the three British Nuclear Fuels plc's (BNFL) installations at Sellafield, Springfields and Capenhurst, the two UK Atomic Energy Authority (UKAEA) installations at Harwell and Winfrith, the Ministry of Defence (MOD) installation at Aldermaston, Amersham International plc's installation at Amersham and the eight Central Electricity Generating Board (CEGB) installations at Bradwell, Berkeley, Dungeness, Hinkley, Oldbury, Sizewell, Trawsfynydd and Wylfa (see Figure 1). All the installations operated by BNFL, UKAEA, MOD and Amersham International began to discharge radioactive waste before 1955, with the exception of Winfrith which began to do so in 1964. Seven of the eight CEGB installations began operations between 1961 and 1965 and the eighth, Wylfa, began in 1971. Berkeley and Oldbury have been classed together as they are very near to each other. Other installations, the discharges from which have been at least an order of magnitude less than those from the CEGB installations, for example, Burghfield (Roman *et al.*, 1987), and installations with start-up after the period for which cancer data have been analysed, have been omitted.

Analyses have used only mortality data. These have become progressively less satisfactory since the mid-1960s as indicators of the incidence of some types of cancer and particularly of leukaemia in young people, as treatment has improved and fatality has been reduced. We believe, nevertheless, that local variation in mortality was the best available indicator of local variation in the incidence of leukaemia and of most other cancers during the period of

Correspondence: P.J. Cook-Mozaffari.
Received 9 May 1988, and in revised form, 3 November 1988.

**Figure 1** Location of nuclear installations and installation districts included in the study.

our study (1969–78) as registration of cancer was incomplete and consequently likely to be biased by local interest in local incidence (Swerdlow, 1986; Cook-Mozaffari, 1987).

### Materials and methods

Eleven causes of death (or groups of causes) have been examined separately within the three age bands, 0–24 years, 25–64 years and 65 years and over. These age bands were chosen at the outset of the study, before the data were compiled, to include the band initially examined in the Black (1984) inquiry. The eleven causes are listed in Table I, together with the total number of deaths attributed to them in each age group in the period 1969–78. This limited calendar period was chosen for study for three reasons: (i) to concentrate on the period after the start-up of almost all the installations, when any hazard with a latent period measured in years would be capable of detection; (ii) to be able to examine separately figures for lymphoid leukaemia which, until 1968, had not been possible as deaths from acute lymphoid and acute myeloid leukaemia had been classed together; and (iii) because OPCS were able to make available deaths only for complete quinquennia.

To calculate mortality rates in the pre-1974 LAAs over the period 1959–80, Cook-Mozaffari *et al.* (1987) had first to estimate the mean population in each LAA using data obtained in the 1961, 1971 and 1981 censuses. Figures were already available for each LAA after the earlier two censuses, but new calculations had to be made to build up the populations from data for census wards and enumeration districts to provide comparable figures for 1981. This was possible for the limited number of pre-1974 LAAs studied in the OPCS report, but it was not practicable for us to obtain similar figures for all the 1,316 LAAs throughout the whole country and we have, therefore, been constrained by the need to use the 402 new post-1974 county districts (CDs) as the smallest practicable units for which both 1971 and 1981 census data were available.

Information about cancer deaths is not available by CDs before 1974, but deaths can be summed into pre-1974 LAAs up to 1980. To obtain areal units for which mortality rates could be compared for a period that straddles the 1974 boundary changes we have combined pre-1974 LAAs so as to make them as nearly as possible co-terminous with post-1974 CDs. For 249 districts the correspondence is exact. For the remaining 153 an approximation has been made and in two instances a pair of CDs has had to be combined. The resulting 400 areas – county districts, approximate county districts and combined county districts – are the basic units of this study and are referred to in the remainder of the paper simply as 'districts'.

For each district the population for 1971 by sex and age has been obtained by summation from the 1971 census. A similar population for 1976 has been derived by linear interpolation between the 1971 and 1981 censuses, with the assumption that in 1981 the relative size of the population in the approximate CDs compared with the actual CDs on which each was based was, for each age-sex group, the same as in 1971. This assumption was made possible by the fact that the 1971 census had been published for both the pre-1974 LAAs and the post-1974 CDs. The two sets of figures for 1971 and 1976 were then used to calculate average population figures for the period 1969–78.

The number of deaths that occurred in 1969–78 in each district was obtained in the same way: that is by building up

**Table I** Total number of deaths from selected causes: England and Wales 1969–78 by age

| Cause of death | ICD code[a] | Age 0–24 | 25–64 | 65+ |
|---|---|---|---|---|
| Leukaemia – all types | 204–207 | 4,230 | 10,412 | 16,466 |
| Lymphoid leukaemia | 204 | 2,401 | 2,329 | 6,315 |
| Other leukaemia | 205–207 | 1,829 | 8,083 | 10,151 |
| All lymphomas | 200–202 | 1,628 | 11,882 | 11,818 |
| Hodgkin's disease | 201 | 683 | 4,410 | 2,494 |
| Other lymphomas | 200, 202 | 945 | 7,472 | 9,324 |
| Multiple myeloma | 203 | 3[b] | 4,290 | 8,128 |
| Cancer of lung | 162 | 112[b] | 126,836 | 194,834 |
| Other malignancies | other 140–207 | 6,214 | 278,766 | 523,690 |
| All malignancies | 140–207 | 12,187 | 432,186 | 754,936 |
| All other causes | other | 185,985 | 901,971 | 3,525,583 |
| All causes | 001–999 | 198,172 | 1,334,157 | 4,280,519 |

[a]International Classification of Diseases, 8th Revision (World Health Organization, 1967); [b]These have been grouped with 'other malignancies' in the analyses.

from the allocations to the pre-1974 LAAs. The numbers of deaths expected in each district were then calculated by multiplying the estimated populations by the corresponding 10-year England and Wales mortality rates for each sex and 5-year age group.

*Statistical analysis*

The variation in the observed numbers of deaths in the 400 districts about the numbers expected has been assessed by means of log-linear regression analyses carried out using the GLIM computer program (Payne, 1986). Preliminary analysis showed that for many diseases, including childhood leukaemia and most notably the lymphoid type, the residual variation was in excess of that expected from Poisson sampling theory, and allowance was, therefore, made for extra-Poisson variation using the method of Breslow (1984). In these analyses log-linear models were used to estimate the relative risk (RR) associated with the five variables described below. No account has been taken in this paper of possible variation in mortality associated with external terrestrial gamma-radiation. Preliminary analyses, however, show no evidence of an association. Significance tests were carried out by comparing $\chi^2$ goodness-of-fit statistics as recommended by Breslow (1984), or by comparing estimates of changes in log-relative risks with their standard errors. One-sided tests in the direction of the observed difference were used throughout, apart from tests of heterogeneity which are necessarily many-sided (see also note added in proof at end of paper).

The five variables considered were:

(i) Social class. Data giving the number of persons in each of 15 socioeconomic groupings used in the 1971 census (excluding men and women in the armed forces and in unspecified occupations) have been summed to give for each district the approximate proportion of the population in each of the six principal social-class grades (I, professional; II, intermediate; IIIN, skilled non-manual; IIIM, skilled manual; IV, partly skilled; and V, unskilled) (OPCS, 1978).

(ii) Rural status. Districts have been classified simply as rural and other, using Webber & Craig's (1976) categorisation, and the characteristics of the CDs defined by Webber & Craig have been assumed to apply to the corresponding approximate CDs.

(iii) Population size. Districts have been grouped into those with a population of less than 50,000, 50,000–99,999, 100,000–149,999, 150,000–299,999 and 300,000 and over in 1971.

(iv) Health authority region. Districts have been grouped into the 15 health authority regions to allow for the effects of broad geographical differences and for possible effects of differing diagnostic practice or treatment regimes between health authority regions.

(v) Proximity to a nuclear installation. The position of each nuclear installation has been located from its grid reference on large-scale maps. Circles with a radius that represented 10 miles were drawn around each installation. The proportion of the population falling within this radius was estimated using the method described for the OPCS

*Nuclear installation districts*

In the course of the log-linear regression analyses, RRs have been estimated for all districts that have at least 0.1% of the population within a 10-mile radius, relative to all other districts. Trends in risk with increasing proximity to an installation have been carried out by examining three categories of district: those with 66.0% or more of the population living within 10 miles of an installation (high-proportion zone, 20 districts); those with 10.0–65.9% (middle-proportion zone, 24 districts) and those with 0.1–9.9% within the 10-mile zone (low-proportion zone, 26 districts) (see Figure 1).

In this report the areas considered close to installations are broader than those considered in the OPCS report (Cook-Mozaffari *et al.*, 1987), a condition dictated by the use of CDs as the basis of investigation. The precise relationship between the two sets of areas is set out in Table II. It can be seen that while 98% of the population resident in the high-proportion zone of the present study was included in one of the four zones defined as close to an installation in the OPCS study, this was true of only about 30% of the middle-proportion zone and of less than 1% of the low-proportion zone.

All comparisons in which districts close to grouped installations are examined have been made separately including and excluding Copeland District, which is the only district with more than 0.1% of its population in the vicinity of Sellafield. The results can, therefore, be used to test hypotheses based on the original observations in the vicinity of Sellafield (Black, 1984).

study (Cook-Mozaffari *et al.*, 1987), except that, where no parish had more than half its area within the 10-mile radius, the actual area of the parishes that had any part within the circle was assessed as a percentage of the area of the districts in which they lay, and it was assumed that this percentage of each district's population lay within the 10-mile radius.

### Results

*Socioeconomic and geographical variation*

Districts that are near to nuclear installations were found to have, on average, a higher proportion of their population in social classes I, II and IIIN and a lower proportion in social classes IIIM, IV and V than other districts. These nearby districts also had somewhat smaller populations, very few having more than 150,000 inhabitants. There was little difference in the proportion classed as rural, but a substantial difference in the distribution by regional health authority, 86% (60 out of 70 districts) being located in seven of the 15 regions (North West, Mersey, Oxford, NW Thames, Wessex, South West and Wales).

The variation in RR by these four variables is given in Table III for all leukaemia and the leukaemia sub-types at

**Table II** Relationship between the populations defined as living close to installations in the OPCS study (Cook-Mozaffari *et al.*, 1987) and in the present study (populations in thousands)

| *Zones of the present study (characterised by the percentage of the population resident within 10 miles)* | *Discrete distance zones in the OPCS study* | | | | *Control LAAs in the OPCS study* | *Other LAAs not in the OPCS study* | *Total population included in the present study* |
|---|---|---|---|---|---|---|---|
| | *1*[a] | *2*[b] | *3*[c] | *4*[d] | | | |
| | *population in end category* | | | | | | |
| High-proportion zone (66.0+) | 493 | 326 | 865 | 165 | 12 | 23 | 1,884 |
| Middle-proportion zone (10.0–65.9) | 71 | 96 | 213 | 438 | 211 | 1,713 | 2,742 |
| Low-proportion zone (0.1–9.9) | 0 | 0 | 0 | 3 | 318 | 2,741 | 3,062 |
| Other districts (<0.1) | 0 | 0 | 0 | 0 | 2,117 | 39,348 | 41,465 |
| Total population in 1971 | 564 | 422 | 1,078 | 606 | 2,658 | 43,825 | 49,153 |

[a]Two-thirds of the population resident within 6 miles of an installation; [b]Two-thirds of the population resident within 8 miles but excluding those resident in zone 1; [c]Two-thirds of the population resident within 10 miles but excluding those resident in zones 1 or 2; [d]One-third of the population resident within 10 miles but excluding those resident in zones 1, 2 and 3.

**Table III** Variation in relative risk of death from selected causes at ages 0–24 by social class, rural status, population size and hospital region. Relative risks for each variable are calculated after adjustment for the other three

| | | *Leukaemia all types* | *Lymphoid leukaemia* | *Other leukaemia* |
|---|---|---|---|---|
| Social class | I | 1.06 | 1.03 | 1.09 |
| (RRs associated with | II | 1.09 | 1.07 | 1.12 |
| a 5% shift) | IIIN | 1.03 | 1.05 | 1.00 |
| | IIIM | 1.03 | 0.99 | 1.08 |
| | IV | 1.02 | 1.01 | 1.04 |
| | V | 1.00 | 1.00 | 1.00 |
| *P* value for heterogeneity | | 0.07 | 0.07 | 0.35 |
| *P* value for trend[a] | | 0.001 | 0.001 | 0.24 |
| Rural/other (1.00) | | 1.09 | 1.07 | 1.11 |
| *P* value for difference[a] | | 0.22 | 0.50 | 0.28 |
| Population size | <50 | 1.00 | 1.00 | 1.00 |
| (thousands) | 50–100 | 1.14 | 1.23 | 1.04 |
| | 100–150 | 1.18 | 1.23 | 1.13 |
| | 150–300 | 1.22 | 1.22 | 1.22 |
| | >300 | 1.18 | 1.14 | 1.24 |
| *P* value for heterogeneity | | 1.00[b] | 1.00[c] | 1.00[d] |
| *P* value for trend[a] | | 0.50[e] | 0.24 | 0.50[f] |
| Hospital region | Northern | 1.06 | 1.16 | 0.94 |
| (England and | Yorkshire | 0.93 | 0.89 | 0.98 |
| Wales = 1.00) | North West | 1.01 | 1.16 | 0.84 |
| | Mersey | 1.07 | 1.09 | 1.05 |
| | Trent | 1.04 | 1.09 | 0.98 |
| | W. Midlands | 1.13 | 1.18 | 1.06 |
| | E. Anglia | 0.97 | 1.02 | 0.92 |
| | Oxford | 1.16 | 1.17 | 1.15 |
| | N.W. Thames | 0.96 | 0.81 | 1.18 |
| | N.E. Thames | 0.97 | 0.96 | 0.99 |
| | S.E. Thames | 1.01 | 1.00 | 1.04 |
| | S.W. Thames | 1.00 | 1.02 | 0.96 |
| | Wessex | 0.90 | 0.83 | 1.02 |
| | South West | 0.90 | 0.87 | 0.95 |
| | Wales | 0.94 | 0.88 | 1.01 |
| *P* value for heterogeneity | | 0.50 | 0.02 | 0.93 |

[a]One-sided tests; [b] deviance based *p*-value 0.40; [c]deviance based *p*-value 0.45; [d]deviance based *p*-value 0.23; [e]deviance based *p*-value 0.10; [f]deviance based *p*-value 0.01 (see noted added in proof at end of paper).

ages 0–24. For all leukaemia there is an upward trend in risk with an increasing proportion of the population being of higher social class after adjustment for the other three variables ($P=0.001$) but the effect is confined to lymphoid leukaemia ($P=0.001$) and is not present for other types ($P=0.24$). For lymphoid leukaemia also there is evidence of regional variation ($P=0.02$). It should be noted that the estimated RRs associated with social class in Table III describe the relationship between mortality and the social-class structure of the districts. They do not describe the risk to an individual associated with belonging to a particular social class, but the ratio of the RRs for any two social classes shows instead the overall effect on mortality in a district associated with a shift of 5% of the total population from the social class of the denominator to that of the numerator, when the proportions in the other classes remain the same. For example, the results for leukaemia of all types would indicate that a 3% shift from social class IV to social class I would tend to increase the number of deaths observed in the district by a factor of $(1.06/1.02)^{3/5}$.

### *Variation in risk in the vicinity of nuclear installations*

Table IV gives the RRs at ages 0–24 and 25–64 for districts that have 0.1% or more of their population living within 10 miles of an installation. Values are given both with and without adjustment for the socioeconomic and geographical variables and both including and excluding Copeland District. At ages 0–24 there is a tendency for the RRs to be slightly higher and for the significance levels to be slightly more extreme with the inclusion of Copeland, but the effect is small. At other ages the exclusion of Copeland makes practically no difference. In the following description of results reference is, therefore, made only to data including Copeland district.

Elevated RRs occur at ages 0–24 for all leukaemia and for lymphoid leukaemia. The RRs are highest for lymphoid leukaemia and slightly higher after adjustment than before (values after adjustment: all leukaemia RR = 1.15, $P=0.01$; lymphoid leukaemia RR = 1.21, $P=0.01$). Elevated RRs also occur for all malignancies and Hodgkin's disease that are significant after adjustment (RR = 1.07, $P=0.03$ and RR = 1.24, $P=0.05$ respectively). For non-malignant diseases there is a slight depression of RR which is less marked after adjustment, but which still remains significantly low (RR = 0.97; $P=0.02$).

At ages 25–64, no RRs are significantly raised. For lymphoid leukaemia, the RR is low after adjustment for the background variables (RR = 0.86; $P=0.05$). For all other groups of disease the RRs are close to, and not significantly different from, unity after adjustment (range of RRs 0.97–1.04).

Calculations as in Table IV have also been made for persons aged 65 and over. When all districts including Copeland were considered, no RRs were significantly above unity either before or after adjustment.

In Table V relative risks are given for those individual types of cancer that in Table IV showed a significant deviation from unity after adjustment. Details are given for all installations, all installations excluding Sellafield (i.e. excluding Copeland District), the four categories of installations that were used in the analyses of the OPCS study, and each of the 15 individual installations. Overall figures are given for all districts with at least 0.1% of their

**Table IV** Relative risk of death from selected causes in districts with 0.1% or more of their population resident within 10 miles of a nuclear installation compared with other districts, by age at death. Unadjusted values are given, and also values adjusted for social class, rural status, population size and Regional Health Authority, both including and excluding Copeland District in which Sellafield is situated

| | | *Ages 0–24* | | | | | *Ages 25–64* | | | |
|---|---|---|---|---|---|---|---|---|---|---|
| | | *All districts (including Copeland)* | | *Excluding Copeland* | | | *All districts (including Copeland)* | | *Excluding Copeland* | |
| | | *Relative risk* | *P value*[a] | *Relative risk* | *P value*[a] | | *Relative risk* | *P value*[a] | *Relative risk* | *P value*[a] |
| Leukaemia – all types | unadj. | 1.12 | 0.005 | 1.11 | 0.008 | unadj. | 0.98 | 0.33 | 0.99 | 0.34 |
| | adj. | 1.15 | 0.01[b] | 1.14 | 0.03[c] | adj. | 0.97 | 0.15 | 0.97 | 0.16 |
| Lymphoid leukaemia | unadj. | 1.16 | 0.007 | 1.16 | 0.01 | unadj. | 0.89 | 0.13 | 0.89 | 0.14 |
| | adj. | 1.21 | 0.01[d] | 1.20 | 0.02[e] | adj. | 0.86 | 0.05 | 0.87 | 0.06 |
| Other leukaemia | unadj. | 1.06 | 0.34 | 1.06 | 0.36 | unadj. | 1.01 | 0.35 | 1.01 | 0.34 |
| | adj. | 1.07 | 0.50 | 1.06 | 0.50 | adj. | 1.00 | 0.49 | 1.00 | 0.46 |
| All lymphomas | unadj. | 1.04 | 0.25 | 1.04 | 0.28 | unadj. | 0.99 | 0.50 | 0.99 | 0.50 |
| | adj. | 1.10 | 0.09 | 1.09 | 0.10 | adj. | 0.99 | 0.39 | 0.99 | 0.42 |
| Hodgkin's disease | unadj. | 1.09 | 0.09 | 1.08 | 0.11 | unadj. | 0.98 | 0.30 | 0.98 | 0.32 |
| | adj. | 1.24 | 0.05 | 1.23 | 0.04 | adj. | 0.99 | 0.44 | 1.00 | 0.49 |
| Other lymphomas | unadj. | 1.01 | 0.50 | 1.00 | 0.50 | unadj. | 0.99 | 0.50 | 0.99 | 0.50 |
| | adj. | 1.00 | 0.50 | 0.99 | 0.50 | adj. | 1.00 | 0.49 | 1.00 | 0.50 |
| Multiple myeloma | | – | – | – | – | unadj. | 1.05 | 0.13 | 1.04 | 0.15 |
| | | | | | | adj. | 1.04 | 0.21 | 1.04 | 0.24 |
| Cancer of the lung | | – | – | – | – | unadj. | 0.95 | 0.02 | 0.95 | 0.02 |
| | | | | | | adj. | 0.99 | 0.28 | 0.99 | 0.35 |
| Other malignancies | unadj. | 1.00 | 0.50 | 0.99 | 0.44 | unadj. | 0.99 | 0.19 | 0.99 | 0.16 |
| | adj. | 1.03 | 0.27 | 1.02 | 0.31 | adj. | 0.99 | 0.26 | 0.99 | 0.24 |
| All malignancies | unadj. | 1.04 | 0.06 | 1.04 | 0.09 | unadj. | 0.98 | 0.06 | 0.98 | 0.05 |
| | adj. | 1.07 | 0.03 | 1.06 | 0.05 | adj. | 0.99 | 0.21 | 0.99 | 0.23 |
| Other causes | unadj. | 0.95 | 0.003 | 0.95 | 0.003 | unadj. | 0.96 | 0.04 | 0.95 | 0.03 |
| | adj. | 0.97 | 0.02 | 0.97 | 0.02 | adj. | 0.98 | 0.08 | 0.98 | 0.08 |

[a]One-sided tests calculated by the method of Breslow (1984) to allow for extra-Poisson variation; [b]deviance based *p*-value 0.005; [c]deviance based *p*-value 0.009; [d]deviance based *p*-value 0.004; [e]deviance based *p*-value 0.007 (see note added in proof at end of paper).

population resident within 10 miles of an installation (pooled installation districts) and separate figures are given for the high, middle and low-proportion zones, so that the trend in relative risk with increasing proportion of the population living within 10 miles of an installation can be examined.

For all leukaemia at ages 0–24, when the pooled installation districts are considered for the four categories of installations used in the OPCS study, there is a significant increase near Sellafield (RR = 1.85, $P = 0.03$). The RRs for other pre-1955 installations and for all CEGB installations combined are also raised (RRs 1.14 and 1.15 respectively), although only that for the pre-1955 installations is significantly elevated ($P = 0.03$). For none of the three categories with districts in more than one zone is there evidence of a significant trend in RR with increasing proportion of the population living within 10 miles of an installation. When individual installations other than Sellafield are considered, the RR for the pooled installation districts is raised significantly above unity only for Springfields (RR = 1.25; $P = 0.04$).

For lymphoid leukaemia at ages 0–24, the results are similar except that the RRs for the pooled installation districts in all four OPCS groupings are higher than they are for all leukaemia, although the RR is significantly greater than unity only for the pre-1955 installations other than Sellafield. For the individual installations, the RRs for the pooled installation districts around Springfields and Sizewell are significantly raised ($P = 0.009$ and 0.02 respectively). Neither for the four categories nor for the individual installations is there any indication of trend apart from a decrease in risk with increasing proportion of the population near to Bradwell.

For Hodgkin's disease at ages 0–24, when the pooled installation districts are considered for the four categories, there is a raised RR for the CEGB installations (RR = 1.48, $P = 0.03$) but no clear indication of a trend. When the individual installations are considered, there is a raised RR for the CEGB installation at Wylfa (RR = 4.72, $P = 0.01$) and an increase in risk between the only two zones (low and middle-proportion) that are near to Dungeness ($P = 0.02$).

For lymphoid leukaemia at ages 25–64, the RRs for the pooled installation districts are below unity for each of the summary groupings used in the OPCS study and for all but one of the individual installations, although only for Dungeness is the pooled RR significantly low (RR = 0.53, $P = 0.04$). At Dungeness there is a trend of decreasing risk between the two zones that are near to this installation ($P = 0.05$) and at Winfrith and Trawsfynydd there are trends of increasing risk ($P = 0.02$ and $P = 0.01$ respectively).

Further analyses of the type presented in Table V were made for non-malignant diseases at ages 0–24 but showed no significant deviation from unity and no significant trends either for the four categories of installations or for individual installations.

### Discussion

### *Comparison with OPCS study*

For leukaemia of all types and for lymphoid leukaemia, the results of the present study echo and extend those derived from the OPCS study (Cook-Mozaffari *et al.*, 1987; Forman *et al.*, 1987) despite substantial differences in methodology. For example, when installation LAAs with more than two-thirds of their population resident within 6 miles of an installation were compared with their controls during the period for which information was available on leukaemia subtypes, there was a 1.46-fold increase for leukaemia and a 2-fold increase in deaths from lymphoid leukaemia at ages 0–24 (Forman *et al.*, 1987) while, in the present study, after adjustment for four socioeconomic and demographic variables, there is a 15% increase in leukaemia and a 21% increase in lymphoid leukaemia at ages 0–24 in districts that had any part within 10 miles of nuclear installations ($P=0.01$ in both instances). The differences in the size of the observed effects between the two studies are the combined consequence of three major differences in methodology. First, in the present study, all districts in England and Wales with less than 0.1% of their population within 10 miles of an installation have been included as controls, giving a total control population of over 40 million. In the OPCS study, control areas of approximately the same population size as the installation areas were selected, which gave a control group of some two and a half million overall and of only half a million for comparison with the 6-mile distance zone cited above. Secondly, differences in mortality due to region of the country, urban/rural status, population size and social class structure have been taken into account by regression analysis, instead of by selective matching. Thirdly, to obtain data covering the whole of England and Wales, it has been necessary to base the study on larger geographical units, namely the 402 post-1974 county districts of England and Wales, rather than the 1,316 pre-1974 LAAs. The consequence of this is that we have had to consider larger areas surrounding the installations, so that a total population of nearly seven and a half million has been classified as living near a nuclear installation, while in the OPCS study the total 'exposed' population was under three million (see Table II).

A further finding of the OPCS study was that at ages 25–74 there was a deficit of 6% of deaths from lung cancer and a 4% deficit of deaths from all malignancies in LAAs with at least two-thirds of their population resident within 8 miles of installations (Forman *et al.*, 1987). It does not seem likely that living in the vicinity of a nuclear installation can itself protect against the development of cancer, and it was concluded that the deficits of lung cancer and all malignancies in the installation LAAs among adults were likely to have resulted from socioeconomic or other environmental differences between the installation and the control LAAs. In the present study, after adjustment for the four socioeconomic and geographical variables, the relative risks of death from lung cancer and all malignancies at ages 25–64 in the pooled installation districts compared with other districts are both very close to unity (lung cancer RR=0.99, $P=0.28$; all malignancies RR=0.99, $P=0.21$ (one-sided tests)) and we conclude that the regression adjustments, together with the use of the more restricted age-group, 25–64, have been highly successful in eliminating social, economic and environmental differences, other than proximity to a nuclear installation, that may affect cancer mortality. For diseases other than cancer in the present study, the relative risks are also low before adjustment and move close to unity with adjustment, although the deficit for non-malignant diseases at ages 0–24 remains significant (RR=0.97, $P=0.02$).

The fact that the OPCS study, which was able to identify populations living within 6 miles of an installation, gave a higher estimate of relative risk than the present study, which has considered broader geographical areas, might at first sight be thought to imply that the increases are concentrated very close to the installations. Three observations indicate that this may not be so: first, there is no suggestion of increasing trend in relative risk with an increasing proportion of the population near to an installation in the present study (see Table V); secondly, the difference in the number of excess deaths estimated from the two studies implies that the increase may not be confined to the close geographical areas considered in the OPCS study and that the lower rate observed in the present study is not, therefore, merely a dilution effect (the estimated annual number of excess deaths associated with the RR of 1.21 for lymphoid leukaemia in the present study is about 8 per year based on a total of 437 deaths or, if Copeland is excluded, 7 per year based on a total of 430 deaths compared with 1–2 per year based on 44 deaths associated with the 2-fold RR in the OPCS study); and third, the control LAAs in the OPCS study had very low rates compared with the rates in the whole of the standard regions in which they were situated, which may have been at least in part a chance finding.

In the OPCS study, the increase in lymphoid leukaemia in young people appeared to be confined to installations other than Sellafield with start-up date before 1955. In the present study the increase in the same category of installations is confirmed (RR=1.21, $P=0.02$, see Table V) and there is also an increase around Sellafield (RR=1.94) that is significant for all leukaemia (RR=1.85, $P=0.03$) but not specifically for lymphoid leukaemia (RR=1.94, $P=0.06$). The RR for the districts near the combined CEGB installations is of similar size to that around the pre-1955 installations other than Sellafield, but the number of deaths involved is small and the increase does not reach statistical significance either for all leukaemia or for lymphoid leukaemia.

The most significant results relating to leukaemia in Table V are the increases at ages 0–24 in the mid-proportion zone when all installations are combined. In carrying out a more detailed analysis of the districts which contribute to the increase in risk in the mid-proportion zone, it emerged that the large urban conurbation of Liverpool CD makes up a major proportion of the excess risk in the mid-proportion zone round Capenhurst. Seventy-one of the leukaemias at ages 0–24 were from Liverpool CD alone and the adjusted relative risk for leukaemia in this district is 1.68 ($P=0.001$) and 2.25 ($P<0.001$) for lymphoid leukaemia. If Liverpool CD is subtracted from the all installations grouping in Table V, then the relative risk for all leukaemia at 0–24 years falls from 1.19 ($P=0.01$) to 1.12 ($P=0.08$) in the mid-proportion zone and from 1.15 ($P=0.01$) to 1.14 ($P=0.03$) over all zones. For lymphoid leukaemia the corresponding relative risks are 1.19 ($P=0.06$) for the mid-proportion zone and 1.20 ($P=0.02$) over all zones. Liverpool CD is unique among the installation districts considered in this analysis in terms of its population size and social-class composition and the likelihood of some particular hazard in the district remains a possibility. However, this does not materially change the significance of the overall results.

Further differences from the OPCS study are the findings of an increase in Hodgkin's disease in the age group 0–24 (RR=1.24, $P=0.05$) and a deficit in lymphoid leukaemia in the age group 25–64 (RR=0.86, $P=0.05$) in the vicinity of nuclear installations. Neither has been reported previously. In installation districts the deficit of lymphoid leukaemia at ages 25–64 is not correlated inversely with the excess at ages 0–24, nor is there a general inverse correlation between mortality from lymphoid leukaemia in these two age groups when data for all districts are examined. Similarly, when mortality from lymphoid leukaemia and Hodgkin's disease at ages 0–24 are compared, there is no evidence of any correlation between the two, either when all districts are considered or when districts near a nuclear installation are excluded. Both the deficit of lymphoid leukaemia at ages 25–64 and the excess of Hodgkin's disease at younger ages may be the sort of chance finding that must be expected when many age-specific disease groups are examined.

### *Reasons for the excess of leukaemia*

Several explanations of the increase in leukaemia in the vicinity of the nuclear installations are possible. First, it may be due to local environmental pollution by radiation. Against this explanation are the current assessments of annual radiation doses which, with estimates of the risks of leukaemia per unit dose, together imply that the doses received by populations living in the vicinity of nuclear installations are far below those that would cause any detectable increase in incidence (Hughes & Roberts, 1984; Dionian *et al.*, 1987; Stather *et al.*, 1988; Darby & Doll, 1987). The present data, moreover, fail to provide support

**Table V** Relative risks of death from selected types of cancer at different ages in districts with 0.1% or more of their population resident within 10 miles of a nuclear installation compared with other districts, by installation and percentage of the population within 10 miles adjusted for social class, rural status, population size and Regional Health Authority

| | Leukaemia – all types ages 0–24 | | | | | Lymphoid leukaemia ages 0–24 | | | | |
|---|---|---|---|---|---|---|---|---|---|---|
| | | Relative risks for districts with specified percentage of the population resident within ten miles of an installation (number of deaths in parentheses) | | | | | Relative risks for districts with specified percentage of the population resident within ten miles of an installation (number of deaths in parentheses) | | | |
| | All districts with at least 0.1 | 0.1–9.9 | 10.0–65.9 | 66.0[d] | P value for trend[d] | All districts with at least 0.1 | 0.1–9.9 | 10.0–65.9 | 66.0[d] | P value for trend[d] |
| All installations | 1.15[b] | 1.16[a] (295) | 1.19[b] (269) | 1.09 (181) | 0.36 | 1.21[b] | 1.18[a] (165) | 1.29[b] (160) | 1.16 (112) | 0.40 |
| All excluding Sellafield | 1.14[a] | 1.15[a] (295) | 1.18[a] (269) | 1.05 (170) | 0.26 | 1.20[a] | 1.17 (165) | 1.29[b] (160) | 1.12 (105) | 0.45 |
| *OPCS categories:* | | | | | | | | | | |
| BNFL Sellafield | 1.85[a] | – | – | 1.84[a] (11) | – | 1.94 | – | – | 1.94 (7) | – |
| Other pre-1955 installations | 1.14[a] | 1.23[a] (185) | 1.22[a] (183) | 1.02 (151) | 0.09 | 1.21[a] | 1.21 (102) | 1.32[a] (108) | 1.13 (97) | 0.37 |
| UKAEA Winfrith | 0.96 | 0.93 (6) | 0.97 (12) | – | 0.42 | 1.06 | 1.28 (4) | 0.94 (6) | – | 0.27 |
| CEGB installations | 1.15 | 1.09 (104) | 1.18 (74) | 1.42 (19) | 0.09 | 1.20 | 1.12 (59) | 1.32[a] (46) | 1.11 (8) | 0.20 |
| *Individual installations:* | | | | | | | | | | |
| BNFL Sellafield | 1.81[a] | – | – | 1.79[a] (11) | – | 1.87 | – | – | 1.81 (7) | – |
| Springfields | 1.25[a] | 1.47 (39) | 1.32 (22) | 1.14 (39) | 0.26 | 1.47[b] | 1.69 (27) | 1.45 (12) | 1.47[a] (31) | 0.44 |
| Capenhurst | 1.09 | 1.01 (54) | 1.31 (102) | 0.99 (22) | 0.30 | 0.92 | 0.70 (23) | 1.28 (60) | 0.78 (11) | 0.19 |
| Amersham International | 1.18 | 1.27 (67) | 1.19 (47) | 1.06 (51) | 0.15 | 1.26 | 1.36 (37) | 1.25 (26) | 1.15 (29) | 0.26 |
| MOD Aldermaston | 1.11 | 1.39 (25) | – | 0.95 (32) | 0.13 | 1.29 | 1.37 (15) | – | 1.24 (23) | 0.38 |
| UKAEA Harwell | 0.77 | – | 0.82 (12) | 0.67 (7) | 0.32 | 0.90 | – | 1.19 (10) | 0.48 (3) | 0.08 |
| Winfrith | 0.96 | 0.96 (6) | 1.00 (12) | – | 0.50 | 1.09 | 1.30 (4) | 1.01 (6) | – | 0.32 |
| CEGB Bradwell | 1.17 | 1.62 (9) | 1.05 (19) | 0.98 (3) | 0.14 | 1.02 | 1.72 (6) | 0.96 (10) | 0.00 (0) | 0.04 |
| Berkeley & Oldbury | 1.12 | 1.03 (43) | 1.13 (23) | 1.62 (8) | 0.17 | 1.06 | 0.92 (22) | 1.24 (15) | 1.13 (3) | 0.37 |
| Hinckley | 1.23 | 1.04 (19) | 2.28 (4) | 1.48 (8) | 0.19 | 1.12 | 0.82 (9) | 2.20 (2) | 1.65 (5) | 0.13 |
| Trawsfynydd | 1.21 | 1.08 (11) | 1.65 (6) | – | 0.17 | 1.46 | 1.36 (7) | 1.81 (3) | – | 0.39 |
| Dungeness | 0.93 | 0.96 (12) | 0.91 (6) | – | 0.39 | 0.97 | 1.11 (8) | 0.75 (3) | – | 0.28 |
| Sizewell | 1.44 | 1.29 (10) | 1.65 (12) | – | 0.30 | 1.88[a] | 1.64 (7) | 2.19[a] (9) | – | 0.29 |
| Wylfa | 0.72 | – | 0.74 (4) | – | – | 1.48 | – | 1.52 (4) | – | – |

| | Hodgkin's disease ages 0–24 | | | | | Lymphoid leukaemia ages 25–64 | | | | |
|---|---|---|---|---|---|---|---|---|---|---|
| All installations | 1.24[a] | 0.91 (33) | 1.82[c] (58) | 0.99 (24) | 0.18 | 0.86[a] | 0.88 (136) | 0.92 (122) | 0.76[a] (72) | 0.25 |
| All excluding Sellafield | 1.23[a] | 0.90 (33) | 1.81[c] (58) | 0.94 (22) | 0.18 | 0.87 | 0.88 (136) | 0.92 (122) | 0.77[a] (70) | 0.28 |
| *OPCS categories:* | | | | | | | | | | |
| BNFL Sellafield | 1.86 | – | – | 1.81 (2) | – | 0.53 | – | – | 0.52 (2) | – |
| Other pre-1955 installations | 1.06 | 0.73 (17) | 1.62[a] (36) | 0.94 (21) | 0.50 | 0.90 | 0.94 (79) | 0.99 (80) | 0.81 (65) | 0.22 |
| UKAEA Winfrith | 1.39 | 0.94 (1) | 1.57 (3) | – | 0.20 | 0.52 | 0.19[a] (1) | 0.74 (6) | – | 0.01[f] |
| CEGB installations | 1.48[a] | 1.13 (15) | 2.10[b] (19) | 0:56 (1) | 0.07 | 0.86 | 0.89 (56) | 0.87 (36) | 0.58 (5) | 0.50 |
| *Individual installations:* | | | | | | | | | | |
| BNFL Sellafield | 1.87 | – | – | 1.74 (2) | – | 0.52 | – | – | 0.50 (2) | – |
| Springfields | 0.89 | 0.81 (3) | 0.30 (1) | 1.39 (7) | 0.13 | 0.88 | 1.03 (16) | 0.70 (9) | 0.90 (20) | 0.36 |
| Capenhurst | 1.27 | 1.11 (9) | 1.64 (18) | 0.88 (3) | 0.36 | 0.83 | 0.91 (25) | 0.99 (42) | 0.51[a] (7) | 0.13 |
| Amersham International | 1.25 | 0.51 (4) | 2.74[b] (15) | 0.91 (7) | 0.32 | 0.98 | 0.94 (32) | 1.17 (24) | 0.84 (23) | 0.37 |
| MOD Aldermaston | 0.67 | 0.42 (1) | – | 0.84 (4) | 0.50 | 0.96 | 0.83 (6) | – | 1.02 (13) | 0.41 |
| UKAEA Harwell | 0.65 | – | 1.33 (2) | 0.00 (0) | 0.07 | 0.78 | – | 1.03 (5) | 0.48 (2) | 0.18 |
| Winfrith | 1.36 | 0.82 (1) | 1.52 (3) | – | 0.21 | 0.53 | 0.19 (1) | 0.74 (6) | – | 0.02[g] |
| CEGB Bradwell | 1.41 | 2.35 (2) | 1.42 (4) | 0.00 (0) | 0.14 | 0.67 | 0.57 (2) | 0.75 (8) | 0.41 (1) | 0.39 |
| Berkeley & Oldbury | 1.22 | 1.19 (7) | 1.55 (4) | 0.00 (0) | 0.50 | 0.98 | 1.09 (26) | 0.95 (12) | 0.34 (1) | 0.21 |
| Hinckley | 0.92 | 0.82 (2) | 0.00 (0) | 1.73 (1) | 0.43 | 0.90 | 0.82 (10) | 1.21 (2) | 0.98 (3) | 0.50 |
| Trawsfynydd | 0.99 | 0.63 (1) | 1.96 (1) | – | 0.20 | 1.37 | 0.87 (8) | 2.68[b] (9) | – | 0.01 |
| Dungeness | 2.14 | 0.57 (1) | 4.50[b] (5) | – | 0.02 | 0.53[a] | 0.72 (5) | 0.18[a] (1) | – | 0.05 |
| Sizewell | 1.57 | 1.61 (2) | 1.50 (2) | – | 0.47 | 0.71 | 0.99 (5) | 0.43 (2) | – | 0.17 |
| Wylfa | 4.72[a] | – | 4.49[b] (3) | – | – | 0.64 | – | 0.65 (2) | – | – |

[a]$0.05 \geqslant P > 0.01$; [b]$0.01 \geqslant P > 0.001$; [c]$P \leqslant 0.001$; [d]One-sided tests; [e]Both Sellafield and Winfrith appear twice in this table for each disease, once under 'OPCS categories' and once under 'individual installations'. In some cases the values under these two entries are different, especially at ages 0–24. This is because the estimates are derived from slightly different multiple regression models. In both models the background variables are included but in one case individual terms for the remaining installations are included, while in the other the remaining installations are grouped into 'other pre-1955' and 'CEGB' installations. In no case are the results obtained under the two models substantially different, although in some instances the significance levels are slightly affected; [f]deviance based $p$-value 0.07; [g]deviance based $p$-value 0.08 (see note added in proof at end of paper).

for this explanation in two ways: no trend in relative risk is observed with increasing proximity to an installation as measured by the trend from low to middle to high-proportion zones (see Table V) and the difference in excess risk between the district round Sellafield and those around the other installations is less than a factor of six (RRs 1.85 and 1.14), whereas the estimated annual doses received by children living in the vicinity of Sellafield are many orders of magnitude greater than those estimated for the other nuclear installations (Stather *et al.*, 1988). Similarly, no unusually high exposure to radioactive discharges has been noted in the discharges from Springfields and Capenhurst that could account for the concentration of high rates in the districts near Springfields or in Liverpool CD.

A second possibility is that the increase in leukaemia in young people associated with proximity to the nuclear installations is attributable to some other factor characteristic of the nuclear industry that might cause a hazard to children via the occupation of parents employed in the installations. This cannot be investigated by geographical studies alone but requires the detailed study of affected individuals and this is now being undertaken by several groups of research workers.

A third possibility is that the districts close to nuclear installations differ from those elsewhere in some other characteristic that is relevant to the aetiology of childhood leukaemia. That this should be so seems unlikely, as the adjustments that have been made for geographical variation in socioeconomic and demographic factors that are known to influence mortality from cancer make the relative risks of death from leukaemia, lymphoma, multiple myeloma, lung cancer, all malignancies and all non-malignant diseases in adults close to unity (RRs all between 0.97 and 1.04) and, despite the large numbers in some instances, not statistically significant. Nevertheless, the causes of different types of cancer differ greatly and it is possible that there is some other factor that influences the incidence of childhood leukaemia that is not allowed for by these adjustments. In this respect the tendency for a higher mortality from leukaemia in young people in districts with relatively high proportions of their populations in social classes I and II (see Table III) deserves further investigation partly because in Seascale, near Sellafield, where an increased mortality from childhood leukaemia was first established, the proportion of the population in social class I was most unusual, namely 47% of the economically active male population in 1971 compared with 5% nationally (Gardner *et al.*, 1987), and partly because it is at odds with mortality data for children based on the social class of their parents which showed no increased risk with social classes I and II (OPCS, 1978). It may be noted that any effect associated with social class appears to operate in the opposite way in Seascale than in Liverpool CD which had a very low proportion of the population in social classes I and II.

Fourth, the observed excess from leukaemia may be due to chance. This seems unlikely, however, as the excess observed in all districts other than Sellafield that were examined to test a hypothesis derived from independent observations on the district near Sellafield was such that as large or larger an excess would have been expected by chance only 3 times in 100. Nevertheless, a 3 in 100 chance may have turned up.

At present there are few data available from other countries with which to compare the increase in leukaemia at ages 0–24 seen in Britain around nuclear installations. Two studies are available from the United States (Crump *et al.*, 1987; Clapp *et al.*, 1987). In the first of these, cancer incidence was related to the Rocky Flats plant in Colorado. No association with the plant was found, but data were not shown separately for children. In the second, an increase of leukaemia was observed in a five-town area of Massachusetts near a nuclear plant in Plymouth. Data were not presented separately by age, but it was reported that the excess was in adults and the elderly.

The results of the present study do not exclude the possibility that some substantial excesses of leukaemia in young people might be found in villages sited very close to the installations, as was observed in Seascale. Detailed investigation of such a possibility is currently being undertaken in our unit and elsewhere.

In the interpretation of these results, it has to be borne in mind that the fatality from leukaemia in young persons was improving throughout the period of the study, the mortality falling from 27.4 per $10^6$ persons aged under 25 years (standardised for age and sex) through 25.1 and 23.3 to 20.3 per $10^6$ per annum in the quinquennia 1961–65 to 1976–80. The period of this reduction was a period when new and more effective treatment was being introduced and the possibility has to be considered that the treatment of patients in the nuclear installation areas was worse than average. Leukaemia in young persons has mostly been treated in regional centres and standardisation for health service region and rural status should have accounted for any major geographical difference in the efficacy of available treatment. Definitive studies need to be based on cancer incidence, but for this purpose standard registration data are inadequate and special efforts need to be made to ensure that coverage is complete.

In future studies of this sort, whether dealing with mortality or incidence data, analyses will need to take account of the fact that national rates may not provide appropriate expected numbers for local studies. This is due not only to the association between disease and the factors such as the four socioeconomic and demographic variables included in the present study, but also those due to further, at present unknown, factors that led to the extra-Poisson variation that we have observed for most of the eleven diseases, including childhood leukaemia and most notably childhood lymphoid leukaemia.

### Conclusion

The results of this study confirm that there has been a small excess mortality from leukaemia and in particular from lymphoid leukaemia in persons aged 0–24 years in districts with some of their population resident within 10 miles of one or other of 15 nuclear installations in England and Wales during the period 1969–78 and suggest that the excess for lymphoid leukaemia in these districts is about 8 deaths a year out of an average annual total of about 240 for England and Wales as a whole. They provide no evidence of any other excess mortality in the installation districts at ages 0–24 years or 25–64 years, except possibly for an increase in Hodgkin's disease in the younger age group. Analysis of the results does not provide any positive evidence that the increase in leukaemia is due to local environmental pollution from the installations.

The small excess mortality from Hodgkin's disease in persons aged 0–24 and the deficiency of lymphoid leukaemia at ages 25–64 years may both be due to random variation.

### *Note added in proof*

Since we completed this paper Professor Breslow has informed us in a personal communication that a recent simulation study has shown significance tests based on a comparison of chi-squared goodness-of-fit statistics, as suggested in Breslow (1984), to perform poorly. We have therefore recalculated all the *p*-values referred to in the paper using a comparison of deviances and also, where the test involves only 1 extra parameter, by comparison of the parameter estimate with its estimated standard error. In all cases the *p*-values calculated by a comparison of deviances were virtually identical to those calculated by comparison of the parameter estimate with its standard error. In the vast majority of cases these were not substantially different from those based on the chi-squared criterion. Three exceptions were: (i) in Table III the test for trend with population size approached significance from leukaemia of all types and became highly significant for leukaemia other than lymphoid leukaemia; (ii) in Table IV the *p*-values for the relative risk of mortality from leukaemia and lymphoid leukaemia at ages 0–24 after adjustment for the four socio-economic variables became smaller, i.e. more highly significant, and (iii) in Table V two of the tests for trend for lymphoid leukaemia at ages 25–64 were no longer significant (see footnotes to Tables).

We are grateful to the National Radiological Protection Board for financial assistance towards this study, to Dr Paul Travers for extensive computing spadework that rendered the OPCS mortality data accessible and to Mrs Sonia Busfield, Mrs Cathy Harwood and Ms Sandra Connell-Hinkes for typing the text and the tables. Detailed data relative to: the identity and characteristics of the installation districts; relative risks for each disease category and age-group by the four socioeconomic and geographical variables; relative risks similar to those shown in Table IV for each disease category at ages 65 and over; relative risks similar to those shown in Table V for other disease categories at ages 0–24 and 25–64 and for all disease categories at ages 65 and over; and relative risks for all leukaemia and for lymphoid leukaemia at ages 0–24 for Liverpool CD and for pooled installation districts excluding Liverpool CD are available on request from Ms Paula Cook-Mozaffari.

### References

BLACK, D. (1984). *Investigation of the Possible Increased Incidence of Cancer in West Cumbria.* HMSO: London.

BRESLOW, N.E. (1984). Extra-Poisson variation in log-linear models. *Appl. Stat.*, **33**, 38.

CLAPP, R.W., COBB, S., CHAN, C.K. & WALKER, B. (1987). Leukaemia near Massachusetts nuclear power plant. *Lancet*, **ii**, 1324.

COOK-MOZAFFARI, P.J. (1987). Cancer near nuclear installations. *Lancet*, **i**, 855.

COOK-MOZAFFARI, P.J., ASHWOOD, F.L., VINCENT, T., FORMAN, D. & ALDERSON, M. (1987). *Cancer Incidence and Mortality in the Vicinity of Nuclear Installations. England and Wales, 1959–1980 (Studies on Medical and Population Subjects, No. 51).* HMSO: London.

CRUMP, K.S., NG, T.-H. & CUDDIHY, R.G. (1987). Cancer incidence patterns in the Denver metropolitan area in relation to the Rocky Flats plant. *Am. J. Epidemiol.*, **126**, 127.

DARBY, S.C. & DOLL, R. (1987). Fallout, radiation doses near Dounreay, and childhood leukaemia. *Br. Med. J.*, **294**, 603.

DIONIAN, J., WAN, S.L. & WRIXON, A.D. (1987). *Radiation Doses to Members of the Public around AWRE, Aldermaston, ROF, Burghfield and AERG Harwell.* NRPB-R202. HMSO: London.

FORMAN, D., COOK-MOZAFFARI, P.J., DARBY, S. & 4 others (1987). Cancer near nuclear installations. *Nature*, **239**, 499.

GARDNER, M.J., HALL, A.J., DOWNES, S. & TERRELL, J.D. (1987). Follow up study of children born to mothers resident in Seascale, West Cumbria (birth cohort). *Br. Med. J.*, **295**, 822.

GARDNER, M.J. & WINTER, P.D. (1984). Mortality in Cumberland during 1959–78 with reference to cancer in young people around Windscale. *Lancet*, **i**, 216.

HUGHES, J.S. & ROBERTS, G.C. (1984). *The Radiation Exposure of the UK Population – 1984 Review.* NRPB-R173. HMSO: London.

OFFICE OF POPULATION CENSUSES AND SURVEYS (1978). *Occupational Mortality 1970–72 Decennial Supplement for England and Wales.* Series DS No. 1. HMSO: London.

PAYNE, C.D. (ed) (1986). *The GLIM System.* Release 3.77. Royal Statistical Society: London.

ROMAN, E., BERAL, V., CARPENTER, L. & 4 others (1987). Childhood leukaemia in the West Berkshire and Basingstoke and North Hampshire District Health Authorities in relation to nuclear establishments in the vicinity. *Br. Med. J.*, **294**, 597.

STATHER, J.W., CLARKE, R.H. & DUNCAN, K.P. (1988). *The Risk of Childhood Leukaemia near Nuclear Establishments.* NRPB-R215. HMSO: London.

SWERDLOW, A.J. (1986). Cancer registration in England and Wales: some aspects relevant to interpretation of the data. *J. R. Stat. Soc. A.*, **149**, 146.

WEBBER, R. & CRAIG, J. (1976). Which local authorities are alike. *OPCS Population Trends*, **5**, 13. HMSO: London.

WORLD HEALTH ORGANIZATION (1967). *International Classification of Diseases. Manual of the International Statistical Classification of Diseases, Injuries, and Causes of Death.* WHO: Geneva.