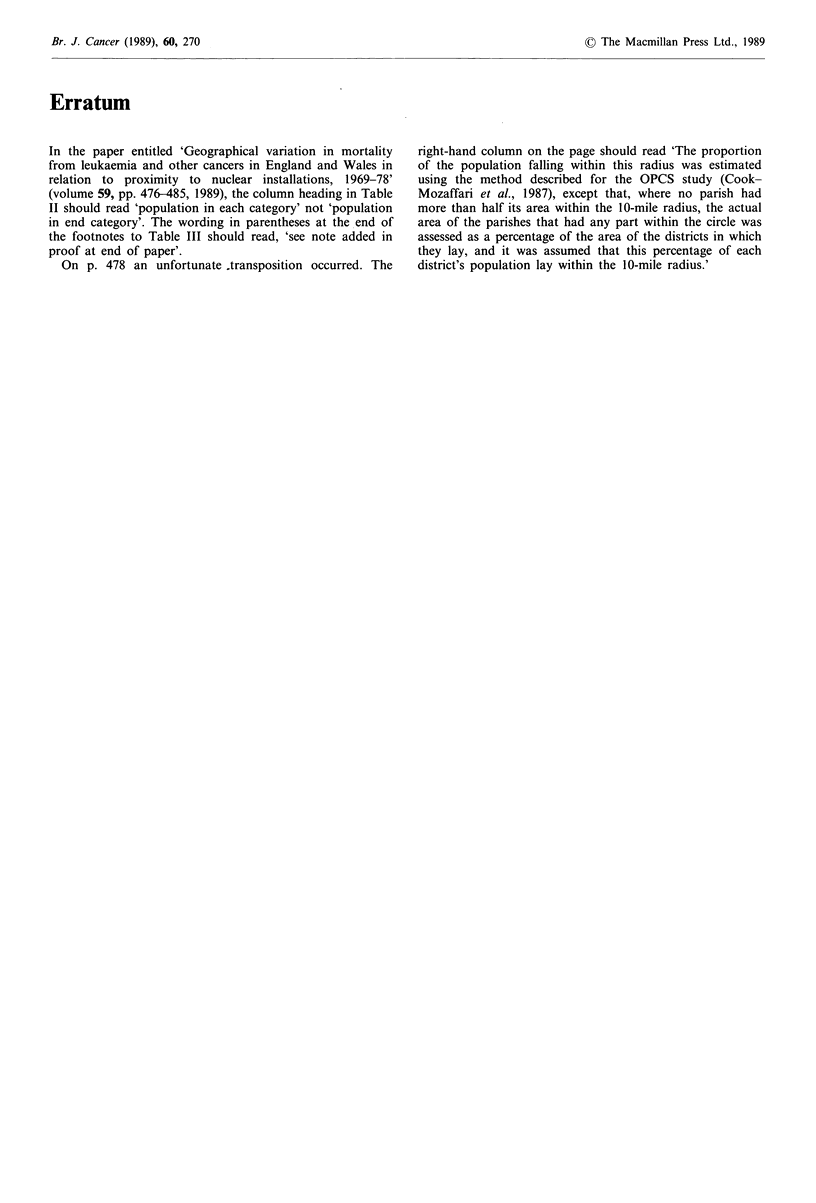# Erratum

**Published:** 1989-08

**Authors:** 


*Br. J. Cancer* (1989), **60**, 270 © The Macmillan Press Ltd., 1989

### Erratum

In the paper entitled 'Geographical variation in mortality from leukaemia and other cancers in England and Wales in relation to proximity to nuclear installations, 1969–78' (volume **59**, pp. 476–485, 1989), the column heading in Table II should read 'population in each category' not 'population in end category'. The wording in parentheses at the end of the footnotes to Table III should read, 'see note added in proof at end of paper'.

On p. 478 an unfortunate .transposition occurred. The right-hand column on the page should read 'The proportion of the population falling within this radius was estimated using the method described for the OPCS study (Cook-Mozaffari *et al.*, 1987), except that, where no parish had more than half its area within the 10-mile radius, the actual area of the parishes that had any part within the circle was assessed as a percentage of the area of the districts in which they lay, and it was assumed that this percentage of each district's population lay within the 10-mile radius.'